# Regorafenib-induced retinal and gastrointestinal hemorrhage in a metastatic colorectal cancer patient with liver dysfunction: A case report: Erratum

**DOI:** 10.1097/MD.0000000000008576

**Published:** 2017-11-03

In the article, “Regorafenib-induced retinal and gastrointestinal hemorrhage in a metastatic colorectal cancer patient with liver dysfunction: A case report”^[1]^, which appeared in Volume 96, Issue 42 of *Medicine*, Dr. Koh-Hei Sonoda's affiliation should have been affiliation f, Department of Ophthalmology, Graduate School of Medical Sciences, Kyushu University, Fukuoka.
